# Supplementary material for: Muscle AMP deaminase activity was lower in Neandertals than in modern humans
Source: Nat Commun. 2025 Jul 10;16:6371. doi: 10.1038/s41467-025-61605-4 (PMC12246493; doi:10.1038/s41467-025-61605-4)
Supplement: Supplementary file 1 — Supplementary Information [file 41467_2025_61605_MOESM1_ESM.pdf]

Supplementary Information for

Muscle AMP deaminase activity was lower in Neandertals than in modern humans

Dominik Macak[1], Shin-Yu Lee[2], Tomas Nyman[3], Henry Ampah-Korsah[3], Emilia Strandback[3], Svante Pääbo[1,2], Hugo Zeberg[1,4]*

Affiliations

[1]Max Planck Institute for Evolutionary Anthropology, Leipzig, Germany

[2]Okinawa Institute of Science and Technology, Onna-son, Japan

[3]Protein Science Facility, Department of Medical Biochemistry and Biophysics, Karolinska Institutet, Stockholm, Sweden

[4]Department of Physiology and Pharmacology, Karolinska Institutet, Stockholm, Sweden

*Corresponding author: hugo.zeberg@ki.se

Supplementary Figures

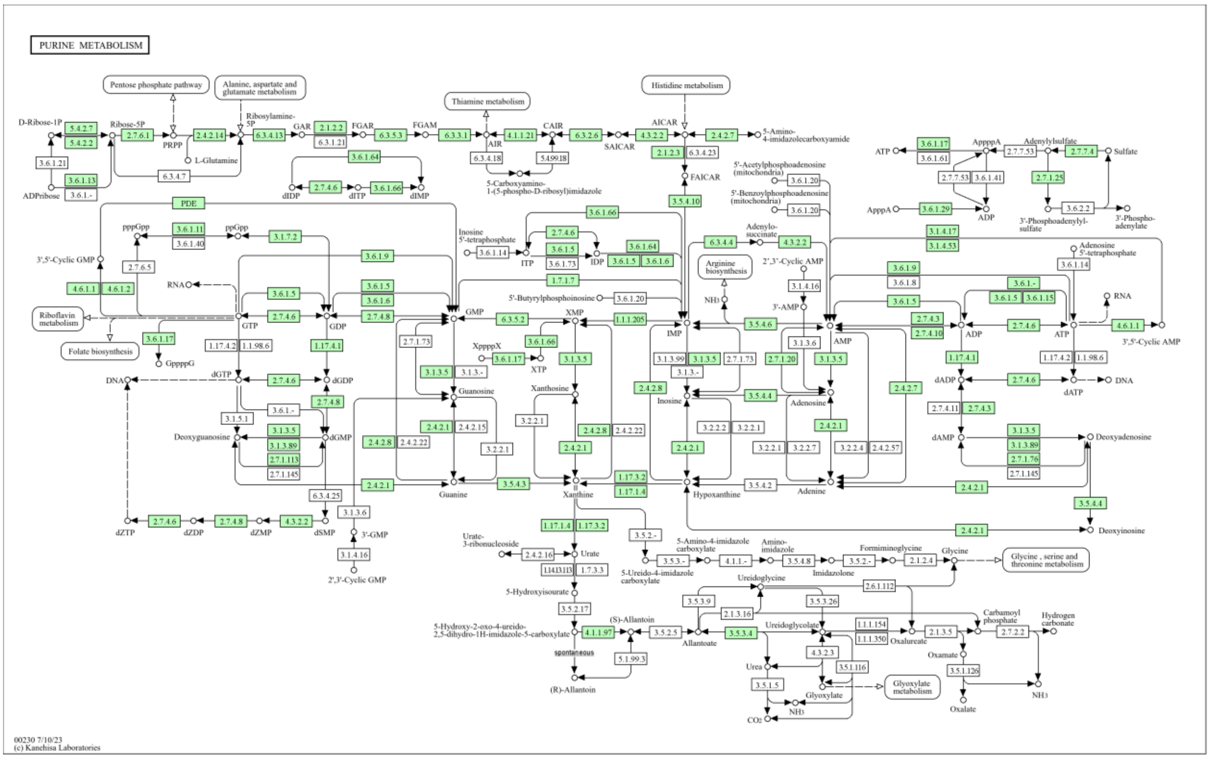

Supplementary Fig. 1 | Purine metabolism pathway in humans. Enzymes and metabolites involved in purine metabolism according to the Kyoto Encyclopedia of Genes and Genomes[1] (KEGG) database map hsa00230 in *Homo sapiens*.

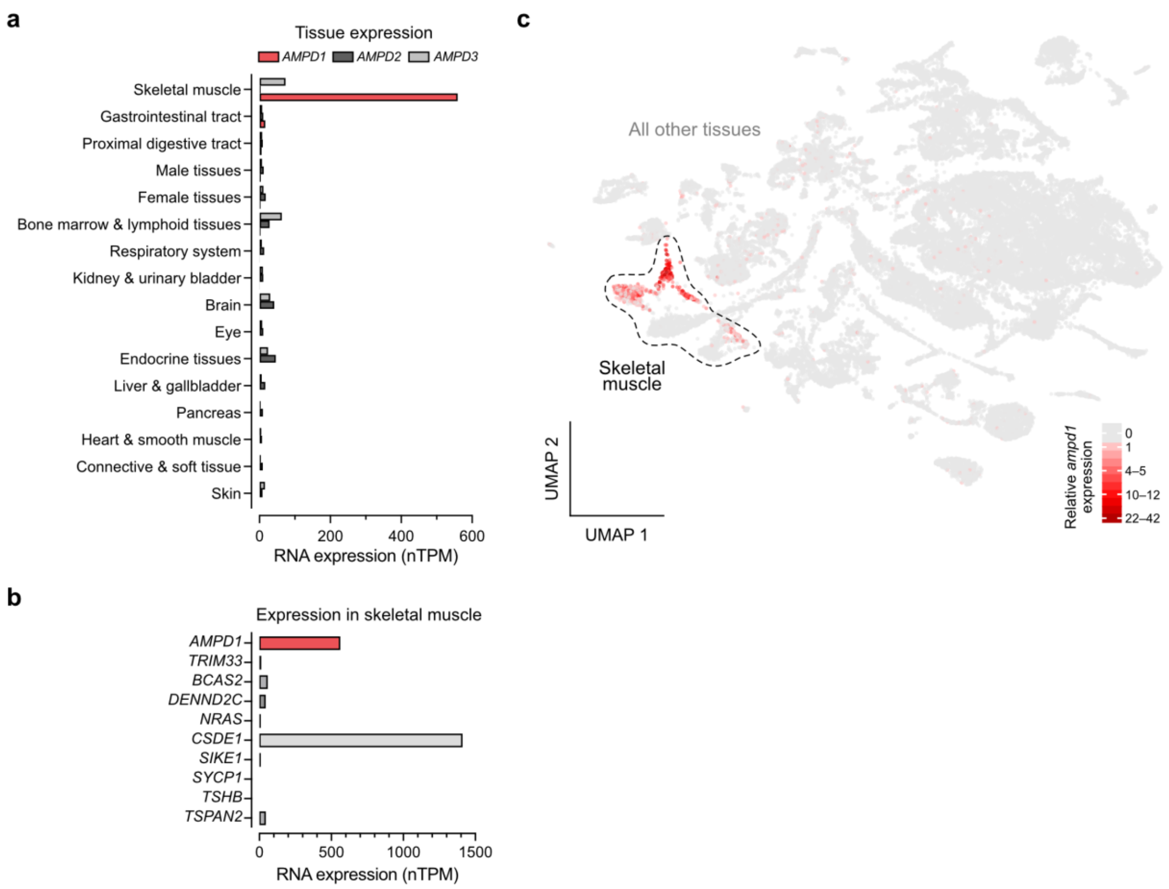

Supplementary Fig. 2 | RNA expression of *AMPD* genes across different tissues. a Tissue mRNA expression of *AMPD1*, *AMPD2*, and *AMPD3*. RNA expression based on transcriptomics data from a consensus dataset (Human Protein Atlas[2] and Genotype-Tissue Expression project[3]). Data shown as normalized number of transcripts per million (nTPM). Skeletal muscle includes tongue. **b** Expression of genes in skeletal muscle, including tongue, at the locus with *AMPD1* (r^2 > 0.6 to rs34526199). **c** Single-cell RNA levels of *ampd1* in 44,102 cells from the developing zebrafish[4]. UMAP plot of 220 identified clusters generated using the UCSC Cell Browser[5]. Skeletal muscle includes fast and slow fibers, and cephalic muscle. Source data are provided as a Source Data file.

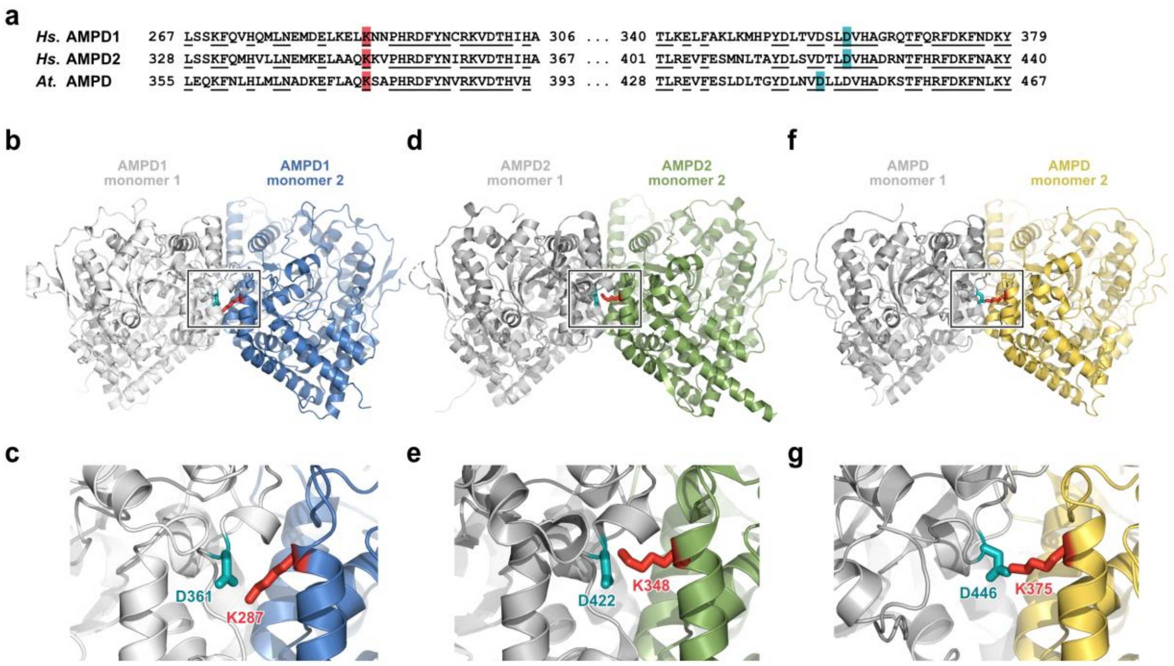

Supplementary Fig. 3 | Conserved Lys–Asp salt bridge at the dimerization interface of AMP deaminases. a Sequence conservation at the positions corresponding to K287 and D361 in human (*Hs.*) AMPD1 (RefSeq NP_000027.3) across human AMPD2 (NP_001355738.1) and *Arabidopsis thaliana* (*At.*) AMPD (NP_565886.1). The conserved lysine and aspartate residues that form the inferred inter-unit salt bridge are highlighted in red and turquoise, respectively. Residues that are completely conserved between these proteins are underlined. **b–g** 3D structures of AMPD dimers showing the interaction at the dimerization interface, with a close-up view of the conserved salt bridge between the lysine (red) and aspartate (turquoise) residues. Human AMPD1 as predicted by AlphaFold[6,7] and modeled onto human AMPD2 (**b, c**), human AMPD2 crystal structure (PDB code 8HU6, ref. [8]) (**d, e**), and *Arabidopsis thaliana* AMPD crystal structure (PDB code 2A3L, ref. [9]) (**f, g**). Notably, in *A. thaliana*, D446 forms the salt bridge with K375 and appears shifted (corresponding to D358 in human AMPD1). Despite this shift, both aspartate residues are conserved across these proteins.

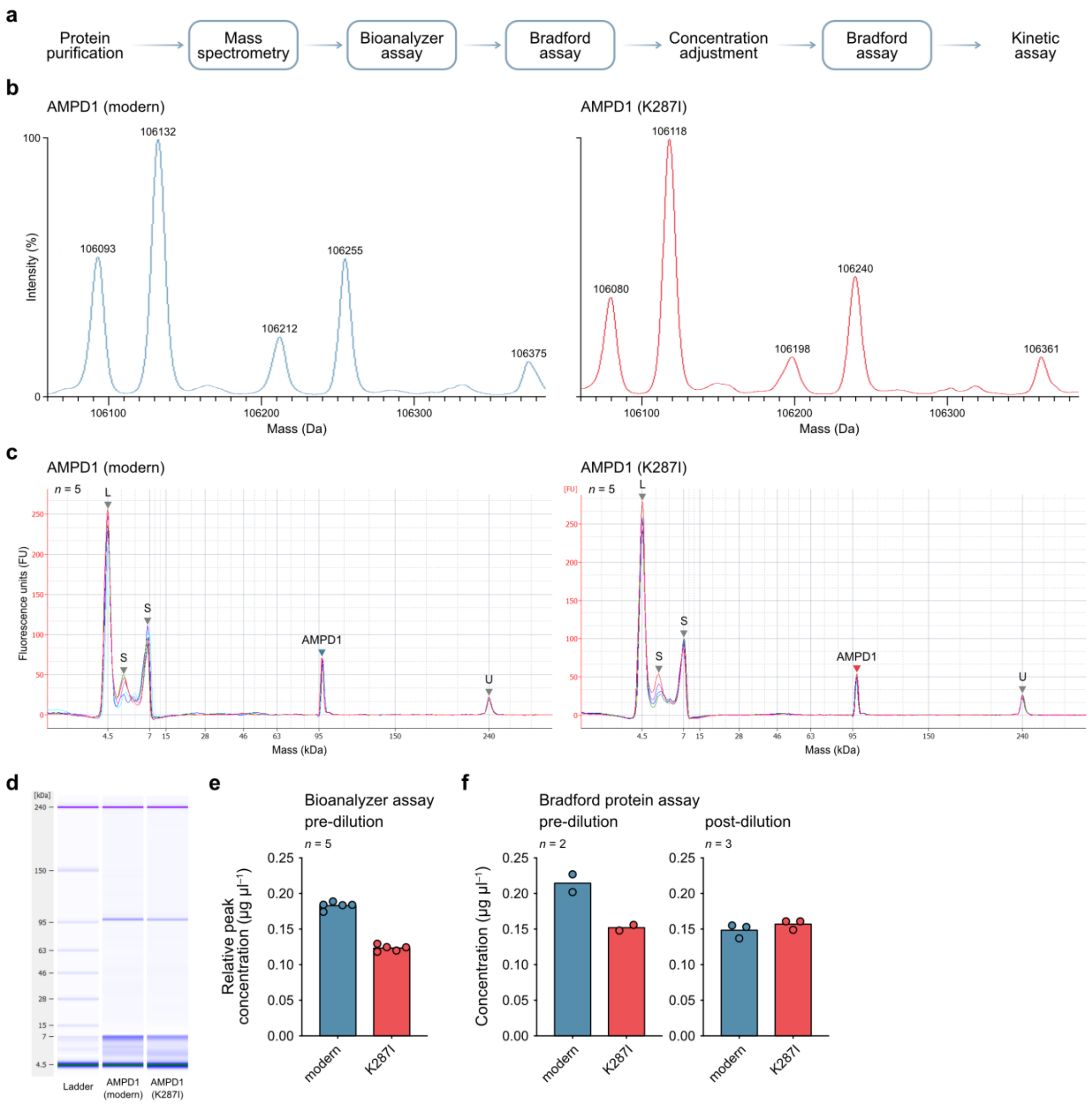

Supplementary Fig. 4 | Quality controls of recombinant AMPD1 proteins. a Quality control assays for recombinant AMPD1 proteins purified from human Expi293F cells. Proteins were analyzed by whole-protein mass spectrometry (MS) to confirm masses, assessed for purity and size using a Bioanalyzer Protein 230 assay, and quantified with Bradford assays. Protein concentrations were adjusted by dilution and re-measured before use in kinetic assays. **b** Whole-protein MS analysis of recombinant modern and Neandertal (K287I) AMPD1 proteins. The calculated masses, accounting for GFP maturation (–21 Da) and N-terminal processing/acylation (–88 Da), are 106134 Da (modern) and 106119 Da (K287I). Detected main peaks deviated by –2 Da and –1 Da, respectively. Additional peaks in 80 Da increments suggest varying phosphorylation levels. A consistent 14 Da difference between the major peaks of the two AMPD1 variants confirms the lysine-to-isoleucine substitution. **c** Electropherograms of recombinant modern AMPD1 and of AMPD1 K287I proteins measured with the Agilent Bioanalyzer Protein 230 assay (*n* = 5 measurements). Both proteins showed a single distinct peak corresponding to a molecular size of 98 kDa. Peak abbreviations: L, lower marker; S, system; U, upper marker. **d** Digital gel electrophoresis images of the recombinant AMPD1 proteins from panel c, with protein sizes indicated in kDa. Representative images of *n* = 5 measurements. **e** Relative peak

concentrations of recombinant AMPD1 proteins from panels c and d, determined by the integrated area under the AMPD1 peak relative to the upper marker peak (*n* = 5 measurements). **f** Bradford assay showing AMPD1 protein concentrations, measured before (*n* = 2 measurements) and after (*n* = 3 measurements) concentration adjustment. Data points represent individual replicates, with bars showing the mean. Source data are provided as a Source Data file.

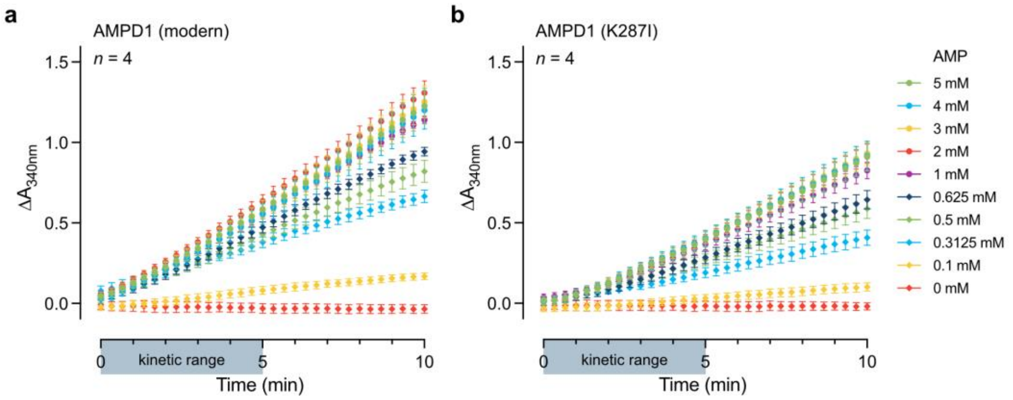

Supplementary Fig. 5 | Kinetic curves of recombinant modern and Neandertal AMPD1 proteins *in vitro*. Recombinant modern (**a**) and Neandertal (K287I) (**b**) AMPD1 proteins were assayed at 0.4 µg of protein per reaction and at 37°C using varying levels of substrate (AMP) by coupling it to the IMP dehydrogenase reaction and measuring the increase in absorbance A at 340 nm over time. Linear regression of independent replicates (n = 4) are shown and used to calculate velocities. The linear kinetic range used to calculate reaction rates is highlighted in gray. Error bars indicate SEM. Source data are provided as a Source Data file.

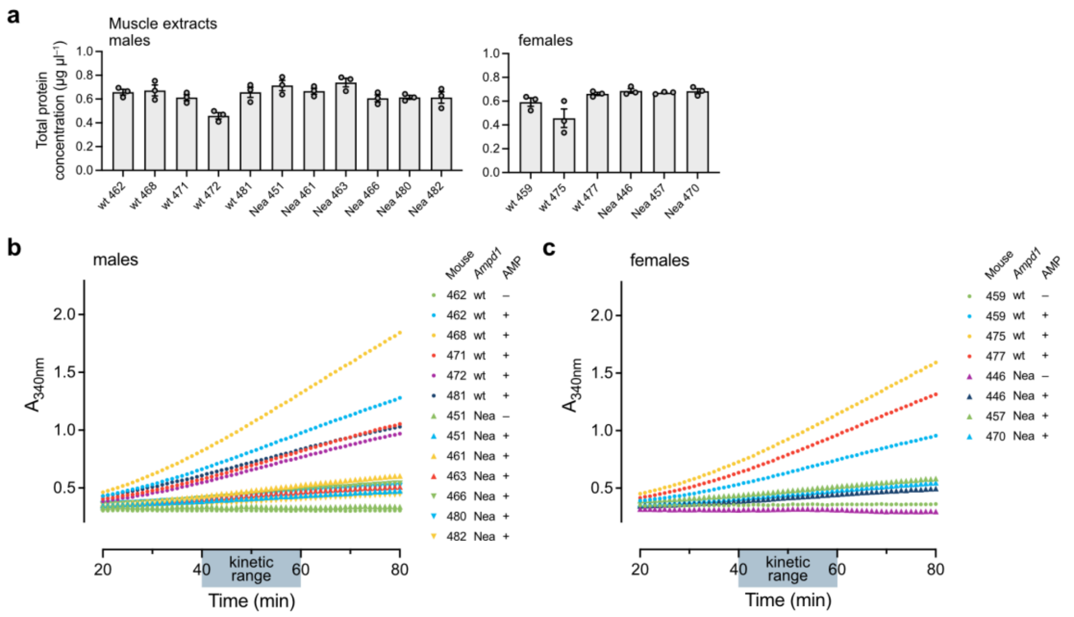

Supplementary Fig. 6 | AMP deaminase activities from protein extracted from *extensor digitorum longus* muscle from transgenic mice carrying the Neandertal mutation in *Ampd1*. **a** Measured total protein from skeletal muscle extracts from transgenic mice carrying the Neandertal (Nea) mutation in *Ampd1* (n = 6 males, 3 females) as well as their wild-type (wt) littermates (n = 5 males, 3 females), determined by Pierce BCA assay. Bars and errors represent the mean ± SEM from n = 3 measurements. Muscle extracts from male (**b**) and female (**c**) mice were assayed at 10 µg total protein per reaction and at 37°C with and without 4 mM AMP and the increase in absorbance A at 340 nm was measured over time. The linear kinetic range used to calculate reaction rates is highlighted in gray. Source data are provided as a Source Data file.

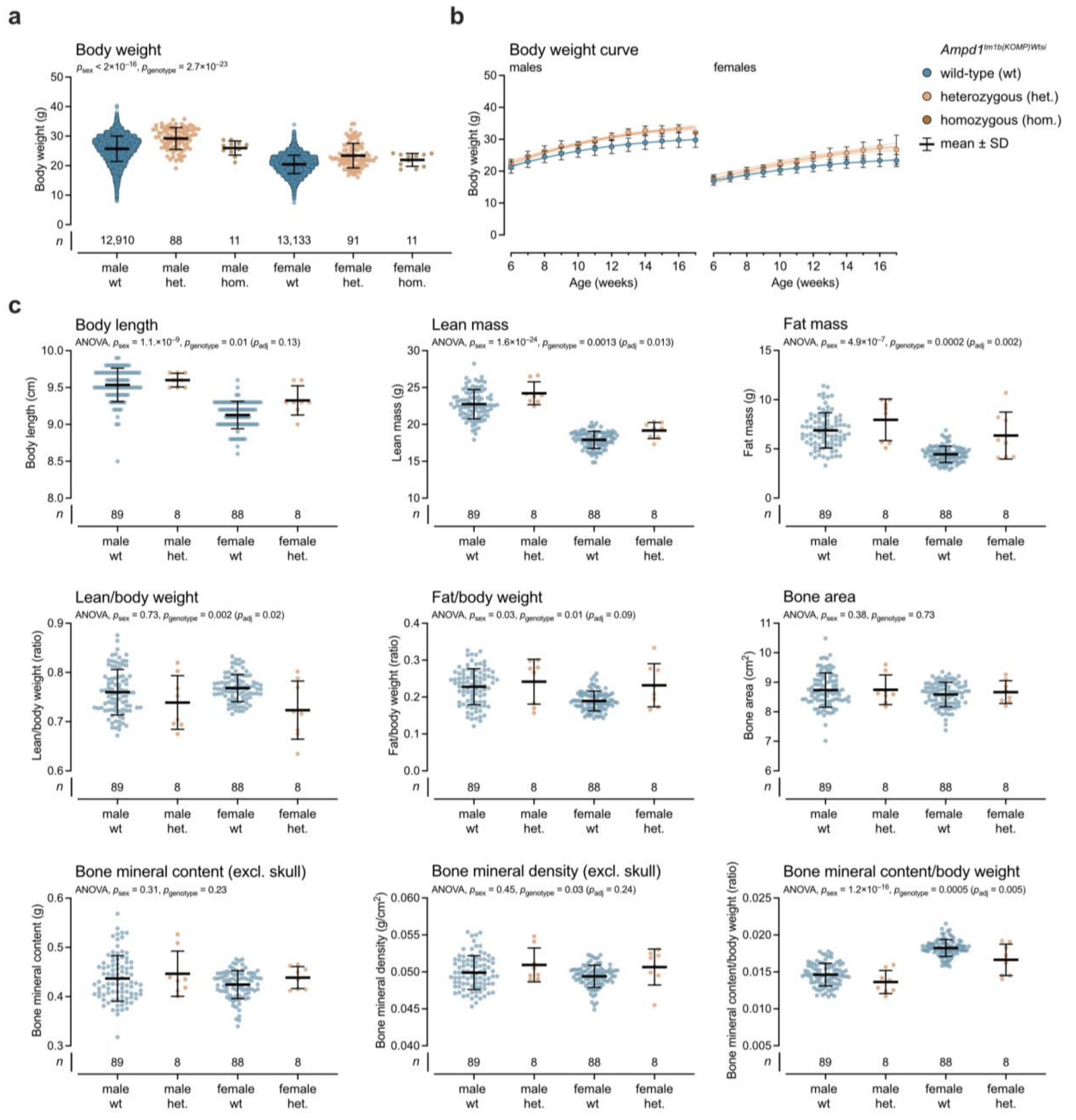

Supplementary Fig. 7 | Phenotypic effects of *Ampd1* knockout mice on body weight and body composition. a Body weight of heterozygous (het.; *n* = 88 males, 91 females) and homozygous (hom.; *n* = 11 males, 11 females) *Ampd1* mutants compared to wild-type controls (wt; *n* = 12,910 males, 13,133 females). Lines and error bars represent mean ± SD. **b** Growth curves of body weight of heterozygous *Ampd1* mutants (*n* = 2 to 28 males, 7 to 23 females) compared to wild-type controls (*n* = 865 to 1,802 males, 701 to 1,959 females) between 6 and 17 weeks of age. Data are fitted to a logistic growth curve, with dots and error bars representing mean ± SD and outer lines 95% CI of fit. **c** Body composition phenotypic assays of heterozygous *Ampd1* mutants (*n* = 8 males, 8 females) compared to wild-type controls (*n* = 89 males, 88 females). All mutants were for the $Ampd1^{tm1b(KOMP)Wtsi}$ allele and all mice were in the early adult life stage. Data from the International Mouse Phenotyping Consortium (IMPC)[10]. Lines and error bars represent mean ± SD. *p* values were calculated by two-way ANOVA, accounting for sex and *Ampd1* genotype, and adjusted for multiple comparisons (p_{adj}) where applicable. Source data are provided as a Source Data file.

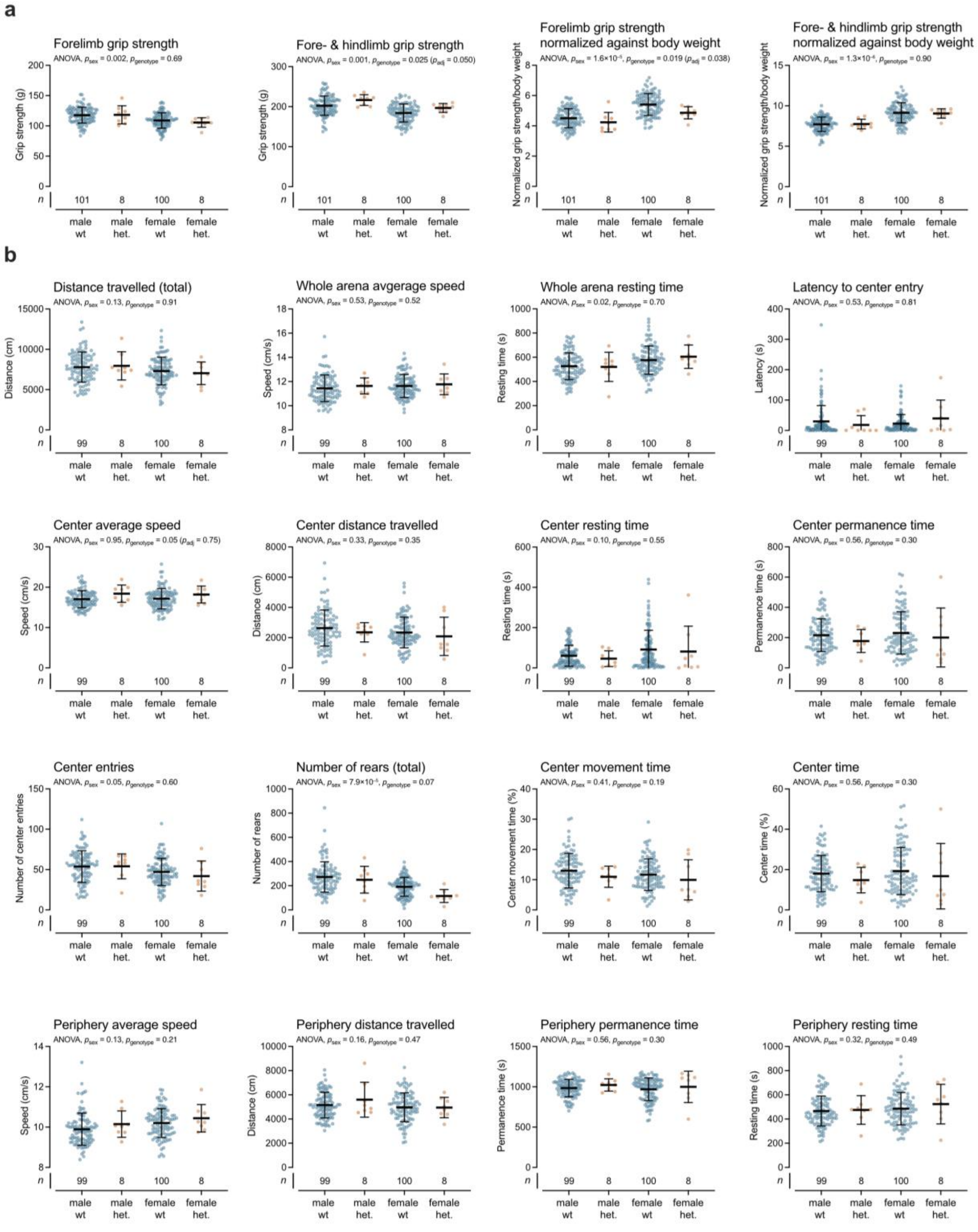

Supplementary Fig. 8 | Phenotypic effects of *Ampd1* knockout mice on grip strength and open field test. a Grip strength (normalized to body weight) of heterozygous *Ampd1* mutants (het.; n = 8 males, 8 females) compared to wild-type controls (wt; n = 101 males, 100 females). **b** Open field phenotypic assay of heterozygous *Ampd1* mutants (n = 8 males, 8 females) compared to wild-type controls (n = 99 males, 100 females). All mutants were for the *Ampd1*$^{tm1b(KOMP)Wtsi}$ allele and all mice were in the early adult life stage. Data from the International Mouse Phenotyping Consortium (IMPC)[10]. Lines and error bars represent mean ± SD. p values were calculated by two-way ANOVA, accounting for sex and *Ampd1* genotype, and adjusted for multiple comparisons (p_{adj}) where applicable. Source data are provided as a Source Data file.

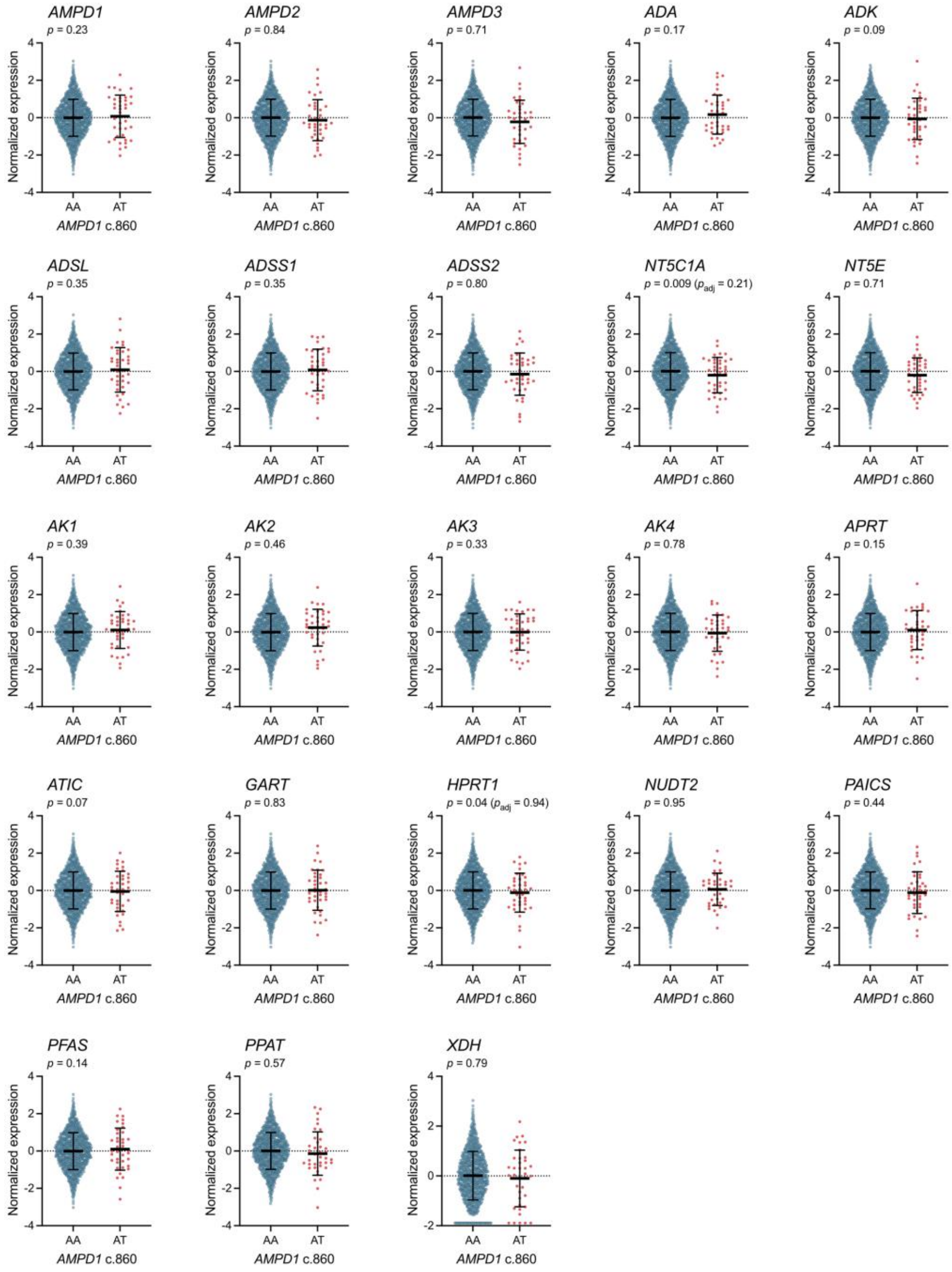

Supplementary Fig. 9 | Expression of genes in AMP metabolism in carriers of the Neandertal allele in *AMPD1*. mRNA expression levels of 23 genes involved in the Gene Ontology category "AMP metabolic process" (GO:0046033) in human skeletal muscle biopsies (GTEx V10, ref. [3]). Data include 778 wild-type (AA) controls and 38 heterozygous (AT) carriers of the Neandertal allele in *AMPD1* (c.860, minor allele frequency: 2.3%). Lines and error bars represent mean ± SD. p values were obtained from expression quantitative trait locus (eQTL) analysis and adjusted (p_{adj}) for multiple comparisons where applicable. Source data are provided as a Source Data file.

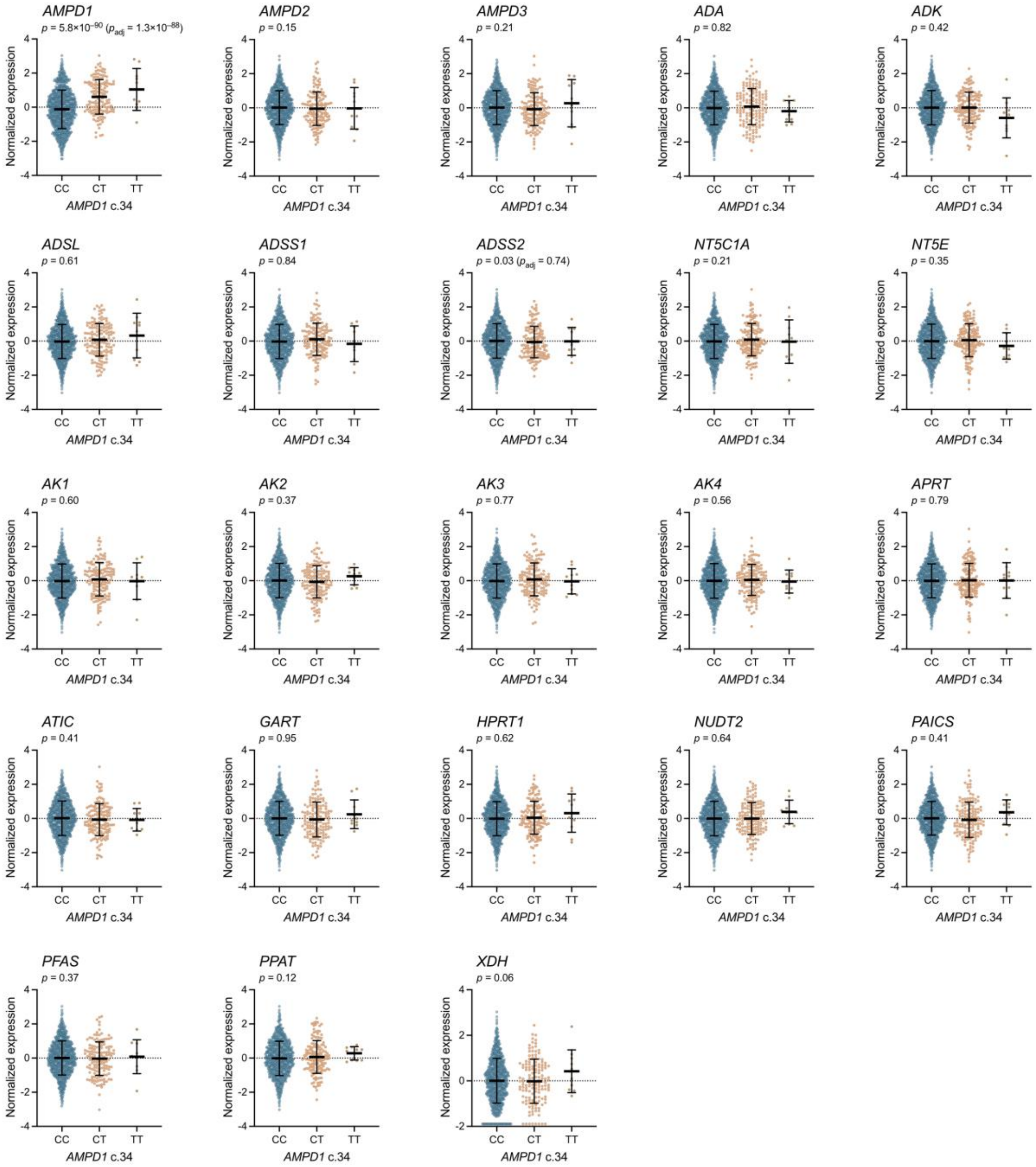

Supplementary Fig. 10 | Expression of genes in AMP metabolism in carriers of the common knockout allele in *AMPD1*. mRNA expression levels of 23 genes involved in the Gene Ontology category “AMP metabolic process” (GO:0046033) in human skeletal muscle biopsies (GTEx V10, ref. [3]). Data include 664 wild-type (CC) controls, and 142 heterozygous (CT) and ten homozygous (TT) carriers of the common knockout allele in *AMPD1* (c.34, minor allele frequency: 9.9%). Lines and error bars represent mean ± SD. p values were obtained from expression quantitative trait locus (eQTL) analysis and adjusted (p_{adj}) for multiple comparisons where applicable. Source data are provided as a Source Data file.

Supplementary Tables

Chr	Position (*hg38*)	Reference allele	Alternative allele	Ancestral allele	Neandertal (fixed in high coverage genomes)	Gene	rsID	Amino acid substitution
**1**	114679616	T	A	T	A	*AMPD1*	rs34526199	K287I
**7**	31823142	T	C	T	C	*PDE1C*	rs61745829	S505G
**17**	10704990	G	A	G	A	*ADPRM*	rs79976834	V22I
**19**	10420933	C	T	C	T	*PDE4A*	rs201432982	R57W

Supplementary Table 1 | Neandertal-derived missense variants in genes involved in purine metabolism. Comparison of the inferred human ancestral allele with the genomes of three high-coverage Neandertals[11-13] across 128 genes involved in purine metabolism, as annotated in the Kyoto Encyclopedia of Genes and Genomes[1] (KEGG pathway hsa00230).

	Common name	Genotype	Allelic depth
**Neandertals (≥5x coverage)**			
Chagyrskaya 8	*Chagyrskaya*	A/A	
Denisova 5	*Altai*	A/A	
El Sidrón 1253	*El Sidrón*	A/A	
Vindija 33.19	*Vindija*	A/A	
**Neandertals (<5x coverage)**			
Gibraltar 1	*Forbes' Quarry*	.	
Goyet Q56-1	Goyet	A	2/2
Grotte Mandrin 1600	*Thorin*	A	1/1
Hohlenstein-Stadel	*HST*	.	
Les Cottés Z4-1514	*Les Cottés*	A	3/3
Mezmaiskaya 1		A	1/1
Mezmaiskaya 2		A	4/4
Scladina I-4A	*Scladina*	.	
Sima de los Huesos	*Sima*	T*	1/1
Spy 94a	*Spy*	A	1/1
**Denisovans**			
Denisova 3	*Denisova*	T/T	
**Neandertal-Denisovan hybrids**			
Denisova 11	*Denny*	T	2/2

Supplementary Table 2 | Genotype calls in various archaic hominin genomes at the position causing the missense mutation in p.K287I in AMPD1. Listed are the bone fragment identifiers, followed by commonly used specimen names. For high-coverage genomes, diploid genotypes are reported; for low coverage genomes (<5x coverage), only a single allele is reported. The allelic depth indicates both the number of reads supporting the called allele and the total number of reads covering the site. Note that *AMPD1* is encoded on the negative strand. Low-coverage genomes were not filtered for deamination. The Sima de los Huesos library suffers from contamination, so the genotype indicated with an asterisk (*) – based on a single read – should be interpreted with caution. Denisova 11 is known to have a Neandertal father and a Denisovan mother[14]. For high-coverage genomes[11-13,15] and the exome-captured El Sidrón genome[16], genotype calls are taken directly from published datasets. For low-coverage genomes, individual reads were called from previous studies[11,14,17-21].

**Phenotype**	***n* (wild-types)**	***n* (mutants)**	**Effect size *d***	***p* (sex)**	***p* (genotype)**	**p_{adj} (genotype)**
**Body weight**	26,043	201	0.6	$<2\times10^{-16}$	2.7×10^{-23}	
**Fat mass**	177	16	1.0	4.9×10^{-7}	0.0002	0.002
**Bone mineral content/body weight**	177	16	–0.9	1.2×10^{-16}	0.0005	0.005
**Lean mass**	177	16	0.9	1.6×10^{-24}	0.001	0.013
**Lean/body weight**	177	16	–0.8	0.73	0.002	0.02
**Fat/body weight**	177	16	0.7	0.03	0.010	0.09
**Body length**	177	16	0.6	1.1×10^{-9}	0.014	0.13
**Bone mineral density (excl. skull)**	177	16	0.6	0.45	0.03	0.24
**Bone mineral content (excl. skull)**	177	16	0.3	0.31	0.23	
**Bone area**	177	16	0.1	0.38	0.73	

Supplementary Table 3 | Phenotypic effects of *Ampd1* knockout mice on body weight and body composition. Phenotypic effects of carrying one copy of the *Ampd1*$^{tm1b(KOMP)Wtsi}$ allele, as shown in Supplementary Fig. 7. Data from the International Mouse Phenotyping Consortium (IMPC)[10]. Effect size calculated as Cohen's *d*. *p* values were calculated using a linear model for body weight, and by two-way ANOVA for other phenotypes, accounting for sex and *Ampd1* genotype, and adjusted for multiple comparisons (p_{adj}) where applicable.

Phenotype	*n* (wild-types)	*n* (mutants)	Effect size *d*	*p* (sex)	*p* (genotype)	p_{adj} (genotype)
**Forelimb grip strength normalized against body weight**	201	16	–0.6	1.6×10^{-5}	0.019	0.038
**Forelimb & hindlimb grip strength**	201	16	0.6	0.001	0.025	0.050
**Center average speed**	199	16	0.5	0.95	0.05	0.75
**Number of rears**	199	16	–0.5	7.9×10^{-5}	0.07	
**Center movement time**	199	16	–0.3	0.41	0.19	
**Periphery average speed**	199	16	0.3	0.13	0.21	
**Periphery permanence time**	199	16	0.3	0.56	0.30	
**Center permanence time**	199	16	–0.3	0.56	0.30	
**Center time**	199	16	–0.3	0.56	0.30	
**Center distance travelled**	199	16	–0.2	0.33	0.35	
**Periphery distance travelled**	199	16	0.2	0.16	0.47	
**Periphery resting time**	199	16	0.2	0.32	0.49	
**Whole arena average speed**	199	16	0.2	0.53	0.52	
**Center resting time**	199	16	–0.2	0.10	0.55	
**Number of center entries**	199	16	–0.1	0.05	0.60	
**Forelimb grip strength**	201	16	–0.1	0.002	0.69	
**Whole arena resting time**	199	16	0.1	0.02	0.70	
**Latency to center entry**	199	16	0.1	0.53	0.81	
**Forelimb & hindlimb grip strength normalized against body weight**	201	16	0.0	1.3×10^{-6}	0.90	
**Distance travelled (total)**	199	16	0.0	0.13	0.91	

Supplementary Table 4 | Phenotypic effects of *Ampd1* knockout mice on grip strength and open field test. Phenotypic effects of carrying one copy of the $Ampd1^{tm1b(KOMP)Wtsi}$ allele, as shown in Supplementary Fig. 8. Data from the International Mouse Phenotyping Consortium (IMPC)[10]. Effect size calculated as Cohen's *d*. *p* values were calculated by two-way ANOVA, accounting for sex and *Ampd1* genotype, and adjusted for multiple comparisons (p_{adj}) where applicable.

Supplementary References

1 Kanehisa, M., Furumichi, M., Sato, Y., Kawashima, M. & Ishiguro-Watanabe, M. KEGG for taxonomy-based analysis of pathways and genomes. *Nucleic Acids Res* **51**, D587-D592 (2023). https://doi.org:10.1093/nar/gkac963

2 Uhlen, M. *et al.* Proteomics. Tissue-based map of the human proteome. *Science* **347**, 1260419 (2015). https://doi.org:10.1126/science.1260419

3 Consortium, G. T. The Genotype-Tissue Expression (GTEx) project. *Nat Genet* **45**, 580-585 (2013). https://doi.org:10.1038/ng.2653

4 Farnsworth, D. R., Saunders, L. M. & Miller, A. C. A single-cell transcriptome atlas for zebrafish development. *Dev Biol* **459**, 100-108 (2020). https://doi.org:10.1016/j.ydbio.2019.11.008

5 Speir, M. L. *et al.* UCSC Cell Browser: visualize your single-cell data. *Bioinformatics* **37**, 4578-4580 (2021). https://doi.org:10.1093/bioinformatics/btab503

6 Jumper, J. *et al.* Highly accurate protein structure prediction with AlphaFold. *Nature* **596**, 583-589 (2021). https://doi.org:10.1038/s41586-021-03819-2

7 Varadi, M. *et al.* AlphaFold Protein Structure Database: massively expanding the structural coverage of protein-sequence space with high-accuracy models. *Nucleic Acids Res* **50**, D439-D444 (2022). https://doi.org:10.1093/nar/gkab1061

8 Kitao, Y. *et al.* The discovery of 3,3-dimethyl-1,2,3,4-tetrahydroquinoxaline-1-carboxamides as AMPD2 inhibitors with a novel mechanism of action. *Bioorg Med Chem Lett* **80**, 129110 (2023). https://doi.org:10.1016/j.bmcl.2022.129110

9 Han, B. W. *et al.* Membrane association, mechanism of action, and structure of Arabidopsis embryonic factor 1 (FAC1). *J Biol Chem* **281**, 14939-14947 (2006). https://doi.org:10.1074/jbc.M513009200

10 Groza, T. *et al.* The International Mouse Phenotyping Consortium: comprehensive knockout phenotyping underpinning the study of human disease. *Nucleic Acids Res* **51**, D1038-D1045 (2023). https://doi.org:10.1093/nar/gkac972

11 Prüfer, K. *et al.* A high-coverage Neandertal genome from Vindija Cave in Croatia. *Science* **358**, 655-658 (2017). https://doi.org:10.1126/science.aao1887

12 Prufer, K. *et al.* The complete genome sequence of a Neanderthal from the Altai Mountains. *Nature* **505**, 43-49 (2014). https://doi.org:10.1038/nature12886

13 Mafessoni, F. *et al.* A high-coverage Neandertal genome from Chagyrskaya Cave. *Proc Natl Acad Sci U S A* **117**, 15132-15136 (2020). https://doi.org:10.1073/pnas.2004944117

14 Slon, V. *et al.* The genome of the offspring of a Neanderthal mother and a Denisovan father. *Nature* **561**, 113-116 (2018). https://doi.org:10.1038/s41586-018-0455-x

15 Meyer, M. *et al.* A high-coverage genome sequence from an archaic Denisovan individual. *Science* **338**, 222-226 (2012). https://doi.org:10.1126/science.1224344

16 Castellano, S. *et al.* Patterns of coding variation in the complete exomes of three Neandertals. *Proc Natl Acad Sci U S A* **111**, 6666-6671 (2014). https://doi.org:10.1073/pnas.1405138111

17 Bokelmann, L. *et al.* A genetic analysis of the Gibraltar Neanderthals. *Proc Natl Acad Sci U S A* **116**, 15610-15615 (2019). https://doi.org:10.1073/pnas.1903984116

18 Hajdinjak, M. *et al.* Reconstructing the genetic history of late Neanderthals. *Nature* **555**, 652-656 (2018). https://doi.org:10.1038/nature26151

19 Meyer, M. *et al.* Nuclear DNA sequences from the Middle Pleistocene Sima de los Huesos hominins. *Nature* **531**, 504-507 (2016). https://doi.org:10.1038/nature17405

20 Peyregne, S. *et al.* Nuclear DNA from two early Neandertals reveals 80,000 years of genetic continuity in Europe. *Sci Adv* **5**, eaaw5873 (2019). https://doi.org:10.1126/sciadv.aaw5873

21 Slimak, L. *et al.* Long genetic and social isolation in Neanderthals before their extinction. *Cell Genom* **4**, 100593 (2024). https://doi.org:10.1016/j.xgen.2024.100593
